# Computed Tomography Predictors of Mortality or Disease Progression in Systemic Sclerosis–Interstitial Lung Disease: A Systematic Review

**DOI:** 10.3389/fmed.2021.807982

**Published:** 2022-01-27

**Authors:** Nicholas Landini, Martina Orlandi, Cosimo Bruni, Edoardo Carlesi, Cosimo Nardi, Linda Calistri, Giovanni Morana, Sara Tomassetti, Stefano Colagrande, Marco Matucci-Cerinic

**Affiliations:** ^1^Department of Radiology, Ca' Foncello General Hospital, Treviso, Italy; ^2^Department of Experimental and Clinical Biomedical Sciences, Azienda Ospedaliero-Universitaria Careggi, University of Florence, Florence, Italy; ^3^Department of Experimental and Clinical Medicine, Division of Rheumatology, Azienda Ospedaliero-Universitaria Careggi and Scleroderma Unit, University of Florence, Florence, Italy; ^4^Department of Experimental and Clinical Medicine, Careggi University Hospital, Florence, Italy; ^5^Unit of Immunology, Rheumatology, Allergy and Rare Diseases, San Raffaele Scientific Institute, Istituti di Ricovero e Cura a Carattere Scientifico, Vita-Salute San Raffaele University, Milan, Italy

**Keywords:** systemic sclerosis (scleroderma), interstitial lung disease (ILD), computed tomography, prognostic factors, mortality, disease progression

## Abstract

**Objective:**

Although interstitial lung disease (ILD) is a major cause of morbidity and mortality in systemic sclerosis (SSc), its prognostication remains challenging. Given that CT represents the gold standard imaging technique in ILD assessment, a systematic review on chest CT findings as predictors of mortality or ILD progression in SSc-ILD was performed.

**Materials and Methods:**

Three databases (Medline, Embase, and Web of Science) were searched to identify all studies analyzing CT mortality or ILD progression predictors in SSc-ILD, from inception to December 2020. ILD progression was defined by worsening of forced vital capacity and/or CT ILD findings. Manuscripts not written in English, with not available full-text, not focusing on SSc-ILD or with SSc-ILD not extrapolated, otherwise with overlap syndromes, pediatric patients, <10 cases or predictors other than CT features were excluded.

**Results:**

Out of 3,513 citations, 15 full-texts (2,332 patients with SSc-ILD) met the inclusion criteria. ILD extent and extensive ILD, ILD densitometric analysis parameters, fibrotic extent and reticulation extent resulted as independent mortality predictors. Extensive ILD is also an independent predictor of death, need for supplemental oxygen or lung transplantation. Honeycombing extent is an independent risk factor for respiratory mortality. Independent predictors of ILD progression were not identified.

**Conclusions:**

ILD extent and extensive ILD independently predict mortality in SSc-ILD on CT, as well as ILD densitometric analysis, fibrotic extent and reticulation extent. Extensive ILD is also a predictor of death, need for supplemental oxygen, or lung transplantation. Honeycombing extent predicts respiratory mortality. CT predictors of ILD progression need to be further investigated.

**Systematic Review Registration:**

https://www.crd.york.ac.uk/prospero/, PROSPERO, identifier: CRD420202005001.

## Introduction

Interstitial lung disease (ILD) is one of the most common complications in systemic sclerosis (SSc) and is a leading cause of mortality (1–3). The screening for ILD is recommended in every patient with SSc (4) and chest CT, as the gold standard imaging technique for ILD assessment, plays a key role in both diagnosis and management (5). Characteristic features of ILD on CT in patients with SSc include ground glass opacities (GGO) and reticulations, with or without bronchiectasis (as signs of fibrosis), configuring a non-specific interstitial pneumonia (NSIP) pattern. Honeycomb lung and usual interstitial pneumonia (UIP) are also possible, though less common in SSc (6). The prevalence of CT-detected SSc-ILD may vary from 47 to 84%, depending on the subtype and the criteria used to define it. The disease course for patients with SSc-ILD is highly variable: it may range from a subclinical disease to a more aggressive phenotype. Thus, the identification of patients with rapidly progressive ILD is crucial to establish a more aggressive therapeutic approach (3). In light of the previously published data (7) on prognostic factors in SSc-ILD, in terms of mortality and disease progression, the aim of our systematic literature review was to collect and discuss available data on chest CT features as prognostic factors in patients with SSc-ILD.

## Materials and Methods

### Data Sources and Search

A systematic search was performed to identify all papers that investigated CT findings as predictor of mortality and/or ILD progression in SSc-ILD. Medline, Embase, and Web of Science databases were searched from the onset of each database to 31 December 2020. Two assessors (N.L. and M.O.) adopted predefined criteria to review all citations. Additionally, references from the selected articles and major reviews were also evaluated. Search terms and strategies are shown in Supplementary Table 1. The systematic review was registered in PROSPERO (https://www.crd.york.ac.uk/prospero/), ID CRD42020205001.

### Study Selection

Both assessors independently reviewed all manuscripts, screening titles and abstracts as first step, then evaluated full texts, looking for studies that identified CT features as predictors of mortality or ILD progression in SSc-ILD. The Preferred Reporting Items for Systematic Reviews and Meta-Analyses (PRISMA) flow diagram (http://prisma-statement.org/prismastatement/flowdiagram.aspx) was adopted. Exclusion criteria were: papers not written in English, no full text available, conditions other than SSc-ILD, works with overlap syndromes or SSc-ILD as part of the cohort but whose data could not be separately extracted, pediatric patients, a study population smaller than 10 patients with SSc-ILD, papers focusing on treatment effects only, studies on predictors other than CT features. ILD progression was defined as ILD radiologic progression in terms of extent or the worsening of forced vital capacity (FVC), as previously proposed in Winstone et al. (7). In addition, FVC decline was considered if the study clearly presented a cut-off to define worsening. There were no restrictions on study quality, design, or follow-up. Disagreements were resolved by consensus or, if not reached, through a third assessor (C.B.).

### Quality Assessment, Data Extraction, Synthesis, and Analysis

The studies' quality was assessed with the Critical Appraisal Skills Program (http://www.casp-uk.net). Quality assessment was based on the method of population sampling, the definitions of SSc and ILD, how the outcome was ascertained and whether confounders were appropriately identified and accounted for in the statistical analysis. Risk of bias and applicability concerns was also evaluated following the Quality Assessment of Diagnostic Accuracy Studies-2 (QUADAS-2) criteria (http://www.bristol.ac.uk/population-health-sciences/projects/quadas/quadas-2/). The two assessors independently evaluated the study quality. Disagreements were resolved by consensus. They extracted the following data from selected papers and recorded them in pre-available forms: study design, subject characteristics and study results. Available anamnestic and clinical data (age, sex, smoking habit, serum auto-antibodies, FVC, and diffusion capacity for carbon monoxide) were recorded and summarized. Hazard ratios and odds ratios for predictors were collected, as well as *p*-values. Due to the heterogeneity of selected papers and results, a formal meta-analysis was not performed.

## Results

### Search Results and Study Characteristics

**Figure 1:**
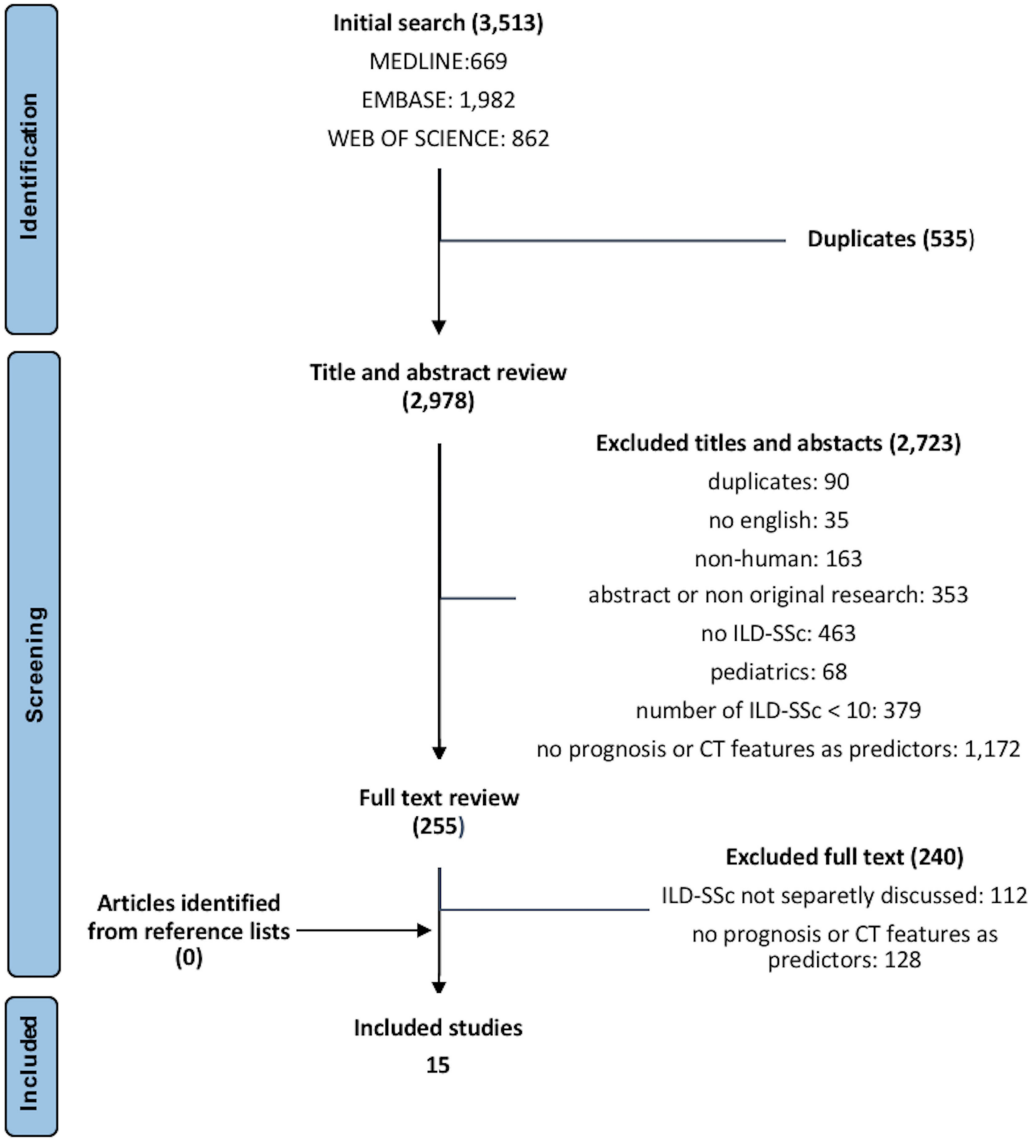
Preferred reporting items for systematic reviews and meta-analyses (PRISMA) diagram of studies' selection. CT, chest computed tomography; ILD, interstitial lung disease; SSc, systemic sclerosis.

Out of 3,513 papers identified, 15 fulfilled inclusion criteria, for a total amount of 2,332 patients with SSc (range 21–595 patients per paper) (8–22). The detailed flow diagram of studies' selection is available in Figure 1. Papers that were not included because only partially meeting inclusion criteria, but relevant for the discussion, are listed in Supplementary Data 1, with the reason of rejection. All included studies were retrospective (8–16, 18–22) or retrospective analyses of prospectively collected data (17) and adopted CT for ILD diagnosis (8–19, 21, 22), except for one that utilized CT or chest X-ray (20). Prone acquisitions were additionally performed to confirm ILD presence in one study (12), while in one work prone scans could be part of the protocol, but only supine CT was analyzed (19). The other studies did not specify. SSc was variously defined, mainly by the 1980 American College of Rheumatology classification criteria (23). The follow-up was heterogeneous, with a mean duration ranging from 3.0 to 11.2 years (10, 12, 15–18) and a median ranging between 4.2 and 12.9 years (8, 9, 13, 14, 19, 21). Two studies followed-up the patients exactly for 1 (22) and 3 (11) years. Studies' quality is summarized in Supplementary Table 2 (Critical Appraisal Skills Program) and Supplementary Figure 1 (QUADAS-2 criteria). Considering patients with available data, mean age ranged from 48 to 67 years, 440/2,069 (21.3%) were men, and 1,546/2,309 (66.9%) were not-smokers. The mean disease duration ranged from 1.1 to 12.2 years in nine studies (9–11, 16–20, 22), the median ranged from 1.0 to 5.0 years in three (12, 14, 15). Anti-centromere antibodies were positive in 306/1,818 (16.8%) patients, and 788/1,974 (39.9%) were positive for anti-topoisomerase I antibodies. FVC ranged from 69.4 to 96.4% predicted and diffusion capacity for carbon monoxide ranged from 31.7 to 75.1% predicted. All characteristics of included studies and patients are summarized in Table 1.

**Table 1:** Studies and patients characteristics.

	**Ando et al. (8)**	**Ariani et al. (9)**	**Champtiaux et al. (10)**	**De Santis et al. (11)**	**Forestier et al. (12)**	**Goh et al. (13)**	**Goh et al. (14)**	
**Study characteristics**								
Enrolment, years	1996–2009	N/R	1998–2013	2003–2005	1991-N/R	1990–1999	1990–1999	
Study design	Retrospective	Retrospective	Retrospective	Retrospective	Retrospective	Retrospective	Retrospective	
Main objective(s)	Assessing the effect of glucocorticoid monotherapy on pulmonary function and survival in patients with SSc-ILD	Investigating SSc-CPFE mortality and compare SSc-CPFE characteristics with other SSc subtypes (ILD, emphysema or neither)	Comparing clinical characteristics, PFT and outcome of SSc-CPFE and other SSc-ILD	Investigating if any BALF characteristic was associated with the risk of ILD progression and worse short-term survival in SSc patients	Describing the evolution of SSc-ILD on serial CT, identifying baseline factors associated with ILD worsening on CT, investigating if the evolution of the PFT was correlated with the evolution of CT.	Identifying the optimal CT extent threshold against mortality	Determining the prognostic value of PFT trends at 1 and 2 years in SSc-ILD	
Recruitment Site (Country)	Japan	Italy	France	Italy	France	England	England	
SSc Diagnostic Criteria	N/R	ACR/EULAR 2013	ACR/EULAR 2013	ACR/ARA 1980	ACR/EULAR 2013-Leroy/Medsger	ACR/ARA 1980	ACR/ARA 1980	
ILD Diagnostic Criteria	CT	CT	CT	CT	CT	CT	CT	
Other Inclusion Criteria	No patients with lacking function data or follow- up period shorter than 6 months	No patients <18 years old or no available follow-up data	No ILD related to another etiology or mixed connective tissue disease	N/R	No patients without available clinical evaluation at baseline and at least one CT scan performed during follow up, no overlap syndrome	No patients with unavailable CT, baseline investigations separated by more than 90 days and no follow-up	No patients' dead within 9 months of follow-up or no serial lung function measurements within the first 12 months	
Follow-up, mean y ± SD	9.8 y^*^	4.2^*^	CPFE 10.1 ± 6.4 ILD^°^ 11.2 ± 7.3	3^§^	5.3 ± 4.9	7.4^*^	12.9^*^	
**Patients characteristics**								
No. with SSc-ILD	71	239	131	73	58	215	162	
Age, mean y ± SD	58.2 ± 13.9	CPFE 57.7 ILD^°^ 59.4	N/R	55.6 ± 12.8	54.5 ± 14.9	49.1 ± 13.0	48 ± 13	
Male (%)	13 (18)	CPFE 16 (59) ILD^°^ 36 (23)	CPFE *n = 36*, 27 (75) ILD^°^*n = 72*, 13 (18)	11 (15)	18 (33)	41 (19)	29 (18)	
Disease duration, mean y ± SD	N/R	CPFE 8.6 ILD^°^ 8.4	CPFE *n = 36*, 3.2 ± 5.4 ILD^°^*n = 72*, 3.1 ± 5.9	8.1± 8.0	1^*^	N/R	3.4^*^	
Not smokers (%)	56 (79)	CPFE 32 (74) ILD ^°^ 174 (89)	CPFE *n = 36*, 6 (17) ILD^°^*n = 72*, 48 (67)	63 (86)	13 (23)	197 (92)	149 (62)	
ACA (%)	N/R	CPFE 5 (12) ILD^°^ 27 (14)	CPFE *n = 36*, 0 (0) ILD^°^*n = 72* 1 (1)	14 (19)	10 (18)	N/R	N/R	
ATA (%)	N/R	CPFE 18 (42) ILD^°^ 113 (58)	CPFE *n = 36*, 15 (42) ILD^°^*n = 72* 41 (57)	40 (55)	38 (68)	N/R	70 (43)	
FVC, %- predicted ± SD	75.1 ± 26.1	CPFE 88 ILD^°^ 91	CPFE *n* = *36*, 78 ± 18 ILD^°^*n* = 72 77 ±20	92.7 ± 22.2	96.4 ± 21.3	78.7 ± 21.4	79.6 ± 21.1	
DLCO, %- predicted ± SD	50.4 ± 16.5	CPFE 48 ILD^°^ 61	CPFE *n* = *36*, 39 ± 13 ILD^°^*n* = 72 75.1 ± 12	53.0 ± 14.4	70.1 ± 25.5	55.1 ± 16.8	56.2 ± 16.6	
**Study/Year**	**Kim et al**. **(****15****)**	**Le Goullec** **et al**. **(****16****)**	**Moore et al**. **(****17****)**	**Moore et al**. **(****18****)**	**Saldana et al**. **(****19****)**	**Sanchez -Cano** **et al**. **(****20****)**	**Takei et al**. **(****21****)**	**Vanaken et al**. **(****22****)**
**Study characteristics**								
Enrolment, years	1990–1999	N/R	N/R	N/R	1997–2018	2006–2014	2006–2016	N/R
Study design	Retrospective	Retrospective	Retro/prospective	Retrospective	Retrospective	Retrospective	Retrospective	Retrospective
Main objective(s)	Assessing the serial changes in parenchymal lung disease on CT, correlated with pulmonary functional changes	Determining if ILD extent on CT is predictive of decline and mortality in SSc-ILD	Determining if ILD extent on CT is predictive of decline and mortality in SSc-ILD	Quantifying change in pulmonary function as a predictor of disease outcome in SSc-ILD	Determining whether CT density-based measurements were more prognostically useful than other measures of outcome in SSc-ILD	Evaluating the clinical characteristics of SSc-ILD patients and analyse the differences according to the SSc subtype, factors associated with impairment of lung function, mortality and causes of death	Evaluating if radiographic fibrosis score is a useful predictor of mortality in SSc-ILD	Describing the prevalence of progressive disease in early SSc patients and evaluating the characteristics of patients at risk of progressive disease
Recruitment Site (Country)	Korea-Japan	France	Australia	Australia	Canada	Spain	Japan	Belgium
SSc Diagnostic Criteria	ACR/ARA 1980 Leroy/Medsger	ACR/ARA 1980 Leroy/Medsger	ACR/ARA 1980-Leroy/Medsger	ACR/ARA 1980-Leroy/Medsger	ACR/EULAR 2013	ACR/ARA 1980-Leroy/Medsger	ACR/EULAR 2013	ACR/EULAR 2013
ILD Diagnostic Criteria	CT	CT	CT	CT	CT	CT or Chest X-ray	CT	CT
Other Inclusion Criteria	No patients without serial CT	No patients without one available PFT performed within 2 months of the initial CT and at least one additional PFT during follow up	N/R	No patients without PFT	No patients with emphysema >10% of total lung volume, CT acquired after lobectomy or pneumonectomy, CT demonstrating an acute non-fibrotic ILD abnormality	No overlap syndrome, undifferentiated connective-tissue disease and mixed connective-tissue disease	No overlap syndrome	No patients with disease duration of more than 36 months, no 42-month follow-up available, no CT and PFT available at baseline
Follow-up, mean y ± SD	3.2	6.4 ± 4.2	3.5 ± 2.9	3.0 ± 1.9	7.2^*^	N/R	4.7^*^	3.5^§^
**Patients characteristics**								
No. with SSc-ILD	40	75	172	262	170	595	48	21
Age, mean y ± SD	N/R	52.0 ± 15.8	55.5 ± 13.0	57.6 ± 11.9	53 ± 13	LC 60 ± 14 DC 52 ± 14 SS 58 ± 17	67	55 ± 13.4
Male (%)	N/R	18 (24.0)	34 (19.8)	42 (16)	31 (18)	85 (14)	15 (31)	11 (52)
Disease duration, mean y ± SD	5^*^	2.8 ± 3.8	10.5 ± 10.1	12.2 ± 10.8	1.1	LC 4.9 ± 7.0 DC 3.6 ± 5.9 SS 2.2 ± 4.4	N/R	1.2 ± 1.0
Not smokers (%)	37 (93)	69 (92)	103 (60)	158 (40)	67 (39)	347 (58)	18 (38)	9 (43)
ACA (%)	N/R	9 (12)	22 (13)	*n* = *260*, 44 (17)	13 (19)	143 (24)	*n* = *47*, 17 (36)	1 (5)
ATA (%)	N/R	41 (55)	56 (78)	*n* = *259*, 82 (32)	52 (39)	198 (33)	*n* = *42*, 11 (26)	13 (62)
FVC, % predicted ± SD	N/R	90.0 ± 19.9	84.1 ± 17.4	82.9 ± 20.8	77 ± 21	LC 78.9 ± 21.9 DC 69.4 ± 21.8 SS 78.4 ± 22.4	*n* = *46*, 82.4	88 ± 20.2
DLCO, % predicted ± SD	62.4 ± 21.7	67.2 ± 23.9	56.2 ± 15.2	53.5 ± 16.9	54 ± 19	LC 41.1 ± 45.2 DC 44.2 ± 36.4 SS 31.7 ± 32.7	*n* = *40*, 60.7	55 ± 17.9

* *Median*.

§ *exact time*.

° *ILD without emphysema. ACR, American College of Rheumatology; ACA, Anti-centromere antibodies; ATA, Anti-topoisomerase antibodies; ARA, Australian Rheumatology Association; BALF, bronchoalveolar lavage fluid; CI, confidence interval; CPFE, combined pulmonary fibrosis emphysema; CT, Chest computed tomography; DLCO, diffusion capacity for carbon monoxide; EULAR, European League Against Rheumatism; FVC, forced vital capacity; ILD, interstitial lung disease; n, available patients (if less than total) y, years; LC, limited cutaneous; DC, diffuse cutaneous; N/R, not reported; PFT, pulmonary function tests; SD, standard deviation; SS, sine scleroderma; SSc, systemic sclerosis*.

### Predictors of Mortality

The definitions of independent predictors were provided in the text. The definitions of all investigated CT predictors are reported in Supplementary Data 2.

#### Univariate Analysis

Univariate analyses were performed on combined pulmonary fibrosis emphysema (CPFE) and UIP patterns as manifestation of ILD against other patterns, ILD extent (also with threshold of 5, 10, 15, 20, 25, 30, 35, and 40%), extensive ILD, ILD grade, ILD densitometric analysis parameters, fibrosis extent, GGO presence, proportion and extent, reticulation presence, coarseness and extent (also with threshold of 5, 10, 15, and 20%), honeycombing presence and extent, traction bronchiectasis severity (9 papers, 1,084 patients) (8–13, 19–22). Extensive ILD was also analyzed with univariate analysis for mortality or need for supplemental oxygen or lung transplantation, as combined outcome, in two studies (17, 18). All studies expressed the risk as hazard ratio, except for one that adopted odds ratio, investigating GGO and reticulation presence (20).

CPFE, compared to ILD without emphysema, was not a significant predictor of mortality in Ariani et al. (9), whereas it was correlated with a higher mortality in Champtiaux et al. (10). UIP did not result as a significant predictor (21). ILD extent and the predefined thresholds from 15 to 40%, were significant mortality predictors (13). Extensive disease was a significant predictor in one (12) out of two studies (12, 22). Extensive ILD was also a significant predictor for death, need for supplemental oxygen, or lung transplantation (17, 18), even when adjusted for extent changes during the follow-up (17). However, this was not the case for ILD grade (22). All ILD quantitative analysis variables, except for skewness, were significant predictors (19). The fibrosis extent >14.2% was identified as the best cut-off for mortality prediction, resulting a significant risk factor (21). GGO presence was not a significant predictor (20), contrarily to GGO extent (11). GGO proportion resulted as a weak protective factor, with a hazard ratio of 0.99 (*p*-value 0.001). Moreover, reticulation coarseness and extent (also with threshold of 5, 10, 15, and 20%) were significant predictors of death (13), but not reticulation presence when was considered alone (20). Honeycombing presence and extent were associated with mortality (8, 11, 13). Lastly, traction bronchiectasis severity (21) was a significant predictor, as well. All univariate analyses are reported in Table 2.

**Table 2:** Predictors of mortality in ILD-SSc, univariate analysis.

**Predictor and Study/Year**	**Outcome**	**Hazard ratio**	**Odds ratio**	**Confidence interval**	***p*** **-value**
**CPFE**					
Ariani et al. (9)	Survival	1.6	-	0.7–3.8	0.1
Champtiaux et al. (10)	Survival	**-**	**-**	**-**	**<0.01**
**UIP**					
Takei et al. (21)	5 y survival	0.79	-	0.27–2.17	0.61
**ILD extent**					
Goh et al. (13)	Mortality	**1.03**	**-**	**1.01–1.04**	**<0.0005**
Goh et al. (13) (>5%)	Mortality	1.77	-	0.81–3.86	0.15
Goh et al. (13) (>10%)	Mortality	1.58	-	0.97–3.57	0.07
Goh et al. (13) (>15%)	Mortality	**2.05**	**-**	**1.30–3.23**	**0.002**
Goh et al. (13) (>20%)	Mortality	**2.48**	**-**	**1.57–3.23**	**<0.0005**
Goh et al. (13) (>25%)	Mortality	**2.31**	**-**	**1.43–3.72**	**0.001**
Goh et al. (13) (>30%)	Mortality	**2.86**	**-**	**1.72–4.76**	**<0.0005**
Goh et al. (13) (>35%)	Mortality	**2.80**	**-**	**1.53–5.15**	**0.001**
Goh et al. (13) (>40%)	Mortality	**2.79**	**-**	**1.38–5.64**	**0.004**
**Extensive ILD**					
Moore et al. (17)^§^	Death, need for supplemental oxygen or lung transplantation	**4.49**	**-**	**2.18–9.23**	**<0.0001**
Moore et al. (17)^§^^*^	Death, need for supplemental oxygen or lung transplantation	**4.69**	**-**	**2.23–9.85**	**<0.0001**
Moore et al. (18)^§^	Death, need for supplemental oxygen or lung transplantation	**-**	**-**	**-**	**0.009**
Forestier et al. (12)^§^	Mortality	**1.28**	**-**	**1.08–1.52**	**0.005**
Forestier et al. (12)^§^^°^	Mortality	**1.33**	**-**	**1.10–1.61**	**0.003**
Vanaken et al. (22)^§^	42 mo Mortality	-	-	-	>0.05
**ILD grade**					
Vanaken et al. (22)	42 mo Mortality	-	-	-	>0.05
**ILD quantitative analysis**					
Saldana et al. (19) (HAA %)	Survival	**1.06**	**-**	**1.02–1.10**	**0.005**
Saldana et al. (19) (Skewness)	Survival	**0.43**	**-**	**0.27–0.68**	**<0.001**
Saldana et al. (19) (Kurtosis)	Survival	**0.82**	**-**	**0.78–0.94**	**0.002**
Saldana et al. (19) (MLA HU)	Survival	**1.07**	**-**	**1.03–1.10**	**<0.001**
Saldana et al. (19) (Δ HAA %)	Survival	**1.22**	**-**	**1.08–1.39**	**0.001**
Saldana et al. (19) (Δ Skewness)	Survival	0.23	-	0.04–1.36	0.11
Saldana et al. (19) (Δ Kurtosis)	Survival	**0.69**	**-**	**0.49–0.98**	**0.04**
Saldana et al. (19)(Δ MLA HU)	Survival	**0.006**	**-**	**1.01–1.03**	**0.006**
**Fibrosis extent**					
Takei et al. (21)	5 y survival	**1.05**	**-**	**1.02–1.07**	**<0.001**
**GGO presence**					
Sanchez Cano et al. (20)	Mortality	-	0.97	0.64–1.48	0.9
**GGO proportion**					
Goh et al. (13)	Mortality	**0.99**	**-**	**0.98–0.99**	**0.001**
**GGO extent**					
De Santis et al. (11)	Mortality	**-**	**-**	**-**	**0.035**
**Reticulation presence**					
Sanchez Cano et al. (20)	Mortality	-	1.34	0.91–1.97	0.142
**Reticulation coarseness**					
Goh et al. (13)	Mortality	**1.15**	**-**	**1.06–1.25**	**0.001**
**Reticulation extent**					
Goh et al. (13)	Mortality	**1.05**	**-**	**1.03–1.07**	**<0.0005**
Goh et al. (13) (>5%)	Mortality	**3.06**	**-**	**1.76–5.32**	**0.15**
Goh et al. (13) (>10%)	Mortality	**2.40**	**-**	**1.52–3.80**	**0.07**
Goh et al. (13) (>15%)	Mortality	**2.78**	**-**	**1.68–4.59**	**0.002**
Goh et al. (13) (>20%)	Mortality	**3.57**	**-**	**1.92–6.67**	**<0.0005**
**Honeycombing presence**					
Goh et al. (13)	Mortality	**1.63**	**-**	**1.00–2.66**	**0.05**
Ando et al. (8)	Survival	**-**	**-**	**-**	**0.01**
De Santis et al. (11) (bilateral)	Mortality	**-**	**-**	**-**	**0.045**
**Honeycombing extent**					
De Santis et al. (11)	Mortality	**-**	**-**	**-**	**0.014**
**TB**					
Takei et al. (21)	5 y survival	**2.52**	**-**	**1.24–5.10**	**0.01**

* *Corrected for CT ILD extent changes over time (data available on 93/172 patients)*.

° *ILD extent assessed during the follow-up*.

§ *ILD >20% or =20% with a FVC < 70% predicted. Bold values correspond to statistically significant predictors. ATA, Anti-topoisomerase antibodies; CKD, chronic kidney failure; CPFE, combined pulmonary fibrosis emphysema; GGO, ground glass opacities; HAA, high attenuation areas; HU, Hounsfield unit; ILD, interstitial lung disease; mo, months; MLA, mean lung attenuation; N/R, not reported; PFT, pulmonary function tests; PH, pulmonary hypertension; SSc, systemic sclerosis; TB, traction bronchiectasis severity; UIP, usual interstitial pneumonia y, years*.

#### Multivariate Analysis and Independent Predictors

A multivariate analysis for the prediction of mortality in SSc-ILD subjects was performed in 8 studies and the potential CT mortality predictors were: CPFE (10), ILD extent (13, 14), extensive ILD (13, 14), ILD quantitative analysis parameters (19), fibrosis extent (21), GGO extent (11), reticulation extent (13), honeycombing presence (8), honeycombing extent (11) and traction bronchiectasis severity (21). Extensive ILD was also investigated as predictor of mortality, need for supplemental oxygen, or lung transplantation (17), as combined outcome. Honeycombing extent was also evaluated for respiratory mortality (11) in one study. Covariates for adjusted analysis were different among studies, although not detailed in two of them (10, 11).

While CPFE (10), GGO extent (11), honeycombing presence (8, 11), and traction bronchiectasis severity (21) did not result as independent predictors on multivariate analysis, other independent factors were identified, with the following definitions:

-**ILD extent**, in one study (13), defined as the mean of ILD percentages scored at 5 levels to the nearest 5%.

-**Extensive ILD**, defined, adopting the same scoring system for ILD extent, as 1) ILD extent >30% or ranging from 10 to 30% with a predicted FVC <70% or 2) ILD extent >20% or =20% with a predicted FVC <70%, investigated in two papers (13, 14). In addition, extensive ILD (>20% or =20% with a predicted FVC < 70%) was also an independent predictor of mortality, need for supplemental oxygen, or lung transplantation, even considering the patients who changed the CT score during the follow-up (17).

-**Fibrosis extent** (cut-off >14.2%), in one work (21), as the mean percentage of lung involvement due to reticulation and/or honeycombing, computed on six levels. Different thresholds of lung involvement were firstly tested, to define the best cut-off as mortality predictor.

-**ILD densitometric analysis**, in one study (19), with the following parameters obtained by software analysis: mean lung attenuation, skewness, kurtosis and the percentage of high attenuation areas. The changes between two serial CT of those parameters for each patient were also evaluated.

-**Reticulation extent**, in one work (13), as the mean of percentages scored at 5 levels, to the nearest 5%.

-**Honeycombing extent**, in one work, specifically addressing respiratory mortality (11), as the mean score computed for each lung lobe (0–5 for each lobe, depending on percentage of involvement).

All independent predictors are reported in Table 3 and all multivariate analyses are shown in Supplementary Table 3.

**Table 3:** Independent predictors of mortality in ILD-SSc, multivariate analysis.

**Predictor and Study/Year**	**Outcome**	**Hazard ratio**	**Confidence interval**	***p*** **-value**	**Variables**
**ILD extent**					
Goh et al. (13)	Mortality	1.03	1.02–1.05	<0.005	Age, sex, smoking status, ground glass proportion, reticulation coarseness
**Extensive ILD**					
Goh et al. (13)^$^	Mortality	3.66	2.25–5.97	<0.005	Age, sex, smoking status, PH
Goh et al. (13)^$^^a^	Mortality	3.02	1.46–6.27	<0.005	Age, sex, smoking status, PH
Goh et al. (13)^$^^b^	Mortality	3.76	1.18–4.34	<0.005	Age, sex, smoking status, PH
Goh et al. (13)^§^^ù^	Mortality	3.39 to 3.82	1.51–7.61 2.03–7.19	<0.005	Age, sex, smoking status, PH
Moore et al. (17)^§^	Death, need for supplemental oxygen or lung transplantation	3.00	1.20–7.51	0.02	FVC, DLCO/VA, age at HRCT, ATA
Moore et al. (17)^§^^*^	Death, need for supplemental oxygen or lung transplantation	3.30	1.10–9.93	0.03	FVC, DLCO/VA, age at PFT, PH, immunosuppressant therapy, ATA
Goh et al. (14)^ç^	15 y survival	3.01	1.90–4.74	<0.0005	FVC, DLCO, FVC/DLCO, KCO, age, sex, treatment status, smoking status, ATA, cutaneous involvement
Goh et al. (14)^ç^	15 y survival	2.30	1.43–3.70	<0.001	KCO, KCO decline^°^, CCD, age, sex, treatment status, smoking status, PH, disease duration^#^
Goh et al. (14)^ç^	15 y survival	2.76	1.66–4.80	<0.0005	KCO, KCO decline^£^, CCD, age, sex, treatment status, smoking status, PH, disease duration^∧^
**ILD quantitative analysis**					
Saldana et al. (19) (Skewness)	Mortality	0.15	0.03–0.84	0.03	age, sex, pack-years, race
Saldana et al. (19) (Kurtosis)	Mortality	0.65	0.46–0.94	0.02	age, sex, pack-years, race
Saldana et al. (19) (Δ HAA %)	Mortality	1.37	1.02–1.83	0.03	age, sex, pack-years, race
Saldana et al. (19) (Δ MLA HU)	Mortality	1.05	1.00–1.11	0.04	age, sex, pack-years, race
Saldana et al. (19) (Δ HAA %)	Mortality	1.36	1.17–1.59	<0.001	age, sex, pack-years, visual fibrosis scores
Saldana et al. (19) (Δ Skewness)	Mortality	0.04	0.01–0.31	0.002	age, sex, pack-years, visual fibrosis scores
Saldana et al. (19) (Δ Kurtosis)	Mortality	0.47	0.30–0.74	<0.001	age, sex, pack-years, visual fibrosis scores
Saldana et al. (19) (Δ MLA HU)	Mortality	1.05	1.03–1.06	<0.001	age, sex, pack-years, visual fibrosis scores
Saldana et al. (19) (Δ HAA %)	Mortality	1.36	1.19–1.55	<0.001	ILD-GAP Index
Saldana et al. (19) (Δ Skewness)	Mortality	0.05	0.01–0.32	<0.001	ILD-GAP Index
Saldana et al. (19) (Δ Kurtosis)	Mortality	0.51	0.35–0.76	<0.001	ILD-GAP Index
Saldana et al. (19) (Δ MLA HU)	Mortality	1.04	1.02–1.05	<0.001	ILD-GAP Index
Saldana et al. (19) (Δ HAA %)	Mortality	1.40	1.22–1.61	<0.001	SADL model
Saldana et al. (19) (Δ Skewness)	Mortality	0.04	0.01–0.27	<0.001	SADL model
Saldana et al. (19) (Δ Kurtosis)	Mortality	0.49	0.32–0.74	<0.001	SADL model
Saldana et al. (19) (Δ MLA HU)	Mortality	1.04	1.03–1.06	<0.001	SADL model
**Fibrosis extent**					
Takei et al. (21)	5 y survival	1.03	1.00–1.06	0.049	Age, sex, CKD, FVC, TB
**Reticulation extent**					
Goh et al. (13)	Mortality	1.05	1.02–1.08	<0.001	Age, sex, smoking status, proportion of ground glass, coarseness of reticulation
**Honeycombing extent**					
De Santis et al. (11)	Respiratory mortality	1.23	1.10–1.44	<0.05	N/R

a *Patients without immediate therapy*.

b *Patients with therapy initiated or continued at presentation*.

$ *ILD extent > 30% and ILD ranging from 10 to 30% with a predicted FVC < 70%*.

§ *ILD > 20% and = 20% with a predicted FVC < 70%*.

ù *data available on 108/215 patients*.

ç *adopted Extensive ILD definition not stated*.

* *corrected for CT disease extent changes over time (data available on 93/172 patients)*.

° *at 12 months*,

£ *at 24 months*.

# *data available on 149 patients*.

∧ *data available on 142 patients. ATA, Anti-topoisomerase antibodies; CCD, composite categorical decline; FVC, forced vital capacity; CKD, chronic kidney failure; DLCO, Diffusion capacity for carbon monoxide; DLCO/VA, DLCO by alveolar volume ratio; FVC, forced vital capacity; GAP, gender, age, physiology; HAA, high attenuation areas; HU, Hounsfield unit; ILD, interstitial lung disease; KCO, carbon monoxide transfer coefficient; MLA, mean lung attenuation; N/R, not reported; PFT, pulmonary function tests; PH, pulmonary hypertension; SADL, smoking history, age, DLCO; SSc, systemic sclerosis; TB, traction bronchiectasis severity; y, years*.

### Predictors of ILD Progression

The predictors of ILD progression were investigated in 3 studies, only through univariate analysis (13, 15, 16). They included ILD extent, extensive ILD, ILD grade, fibrosis coarseness, GGO proportion and extent, irregular linear opacities extent, reticulation proportion and extent, honeycombing presence, traction bronchiectasis severity and emphysema extent (13, 15, 16). According to the included manuscripts, ILD progression was defined as progression of ILD extent on CT vs. stability (15) or FVC decline more than 10% predicted (6, 13). Considered timing intervals were the median delay between the two pulmonary function tests (7.2 months) (16) or the median time to decline (61 months) (13).

Among all the parameters, only ILD grade (13, 16) and reticulation extent (13) were statistically significant predictors of FVC decline (Table 4). None was a predictor of ILD progression on CT. The definitions of all investigated CT predictors are reported in Supplementary Data 2.

**Table 4:** Predictors of ILD progression, univariate analysis.

**Predictor and Study/Year**	**Outcome**	**Hazard ratio**	**Confidence interval**	***p*** **-value**
**ILD extent**				
Goh et al. (13)^∧^	FVC decline > 10%	**1.01**	**1.00–1.02**	**0.03**
Le Goullec et al. (16)*	FVC decline > 10%	**2.68**	**1.21–5.94**	**0.01**
**Extensive ILD**				
Le Goullec et al. (16)*^§^	FVC decline > 10%	0.48	0.22–1.09	0.08
**ILD grade**				
Le Goullec et al. (16)*	FVC decline > 10%	1.39	0.60–3.23	0.43
**Fibrosis coarseness**				
Le Goullec et al. (16)*	FVC decline > 10%	0.92	0.43–2.00	0.84
**GGO proportion**				
Goh et al. (13)^∧^	FVC decline > 10%	1.0	0.99–1.00	0.27
Le Goullec et al. (16)*	FVC decline > 10%	1.06	0.48–2.33	0.88
**GGO extent**				
Kim et al. (15)	CT ILD progression	**-**	**-**	0.335
**Irregular linear opacity extent**				
Kim et al. (15)	CT ILD progression	**-**	**-**	0.464
**Reticulation proportion**				
Le Goullec et al. (16)*	FVC decline > 10%	1.30	0.60–2.83	0.50
**Reticulation coarseness**				
Goh et al. (13)^∧^	FVC decline > 10%	1.06	0.99–1.13	0.11
**Reticulation extent**				
Goh et al. (13)^∧^	FVC decline > 10%	**1.03**	**1.01–1.05**	**<0.005**
**Honeycombing presence**				
Goh et al. (13)^∧^	FVC decline > 10%	1.21	0.79–1.85	0.39
Kim et al. (15)	CT ILD progression	**-**	**-**	0.445
**TB**				
Le Goullec et al. (16)*	FVC decline > 10%	0.78	0.36–1.73	0.55
**Emphysema extent**				
Le Goullec et al. (16)*	FVC decline > 10%	1.38	0.47–4.11	0.55

* *The median delay between the two pulmonary function test was 7.2 months*.

∧ *The median time to decline was 61 months*.

§ *ILD > 20% or = 20% with a FVC < 70% predicted. Bold values correspond to statistically significant predictors. CT, computed tomography GGO, ground glass opacities; ILD, interstitial lung disease; TB, traction bronchiectasis severity*.

## Discussion

Our results identified some CT features mainly related to the extent of disease as independent prognostic factors for mortality. In regards to the **prediction of overall deaths**, **ILD extent** (13) and **extensive ILD** represented independent predictors of mortality (13, 14), the latter also for a combined outcome including death, need for supplemental oxygen or lung transplantation (17). In particular, extensive ILD, among all variables, was the most investigated risk factor (6 studies, 3 with multivariable analyses) (12–14, 17, 18, 22). Thus, in our opinion, it could be confidently incorporated in future clinical trial settings, i.e. adopting the definition of extensive ILD as >20% or =20% with a FVC <70% predicted, that was suggested for clinical practice (13) and verified as independent predictor for both mortality (13) and the aforementioned combined outcome (17). However, these data don't match other studies conducted on connective tissue disease-related ILD (CTD-ILD), where ILD extent was not an independent mortality predictor (24–26). Actually, Vanaken et al. (22), on univariate analysis, did not identify extensive ILD as a significant predictor of death: nevertheless, this result could be related to the small sample of patients with SSc-ILD in their study. Moreover, the **fibrosis extent** resulted as an independent predictor of mortality in one study (21), contrarily to the data reported in another recent paper on patients with CTD-ILD (24). Among the various analysis of reticular features (presence, coarseness, extent), **reticulation extent** was the only independent predictor of mortality in the unique work that investigated this variable (specific thresholds were not tested on multivariate analysis) (13), differently to other studies on patients with CTD-ILD (24–26). Similarly, **honeycombing extent** was an independent risk factor for respiratory death, although not for overall mortality (11), whereas it resulted an independent mortality predictor in two works on CTD-ILD (24, 26).

Presumably, the discrepancies between SSc-ILD and CTD-ILD regarding honeycombing extent as prognostic factors could be explained by the fact that honeycombing is less common in patients with SSc-ILD. In fact, NSIP is the most common ILD pattern and it is usually sustained by reticulation with a lower frequency/extent of honeycombing. On the other hand, in other ILD, such as rheumatoid arthritis, UIP and honeycombing may be more frequent and could have had a major burden in the analysis of a CTD-ILD population (5). However, considering that honeycombing extent may have a prognostic role in respiratory mortality, we believe that the role of CT-ILD features should be investigated for this specific outcome. This could allow to split factors that provide prognostic information regarding mortality more directly caused by ILD from those generically associated to death, as expressions of the systemic disease. Moreover, further studies might confirm the prognostic value of those factors that were, respectively, evaluated only in one study, such as fibrosis extent, reticulation (overall mortality) and honeycombing extent (respiratory mortality).

On the other hand, the potential role of **ILD densitometric analysis**, that provides continuous quantitative parameters based on the software-based images assessments, remains debatable. In fact, histogram-based densitometry parameters resulted as independent risk factors for mortality (19), in particular their changes during the follow-up (Table 3). However, when adjusted for baseline and temporal changes in lung function, these automated data were not independently associated with death: in the opinion of the authors, this suggests that densitometric data may not provide additional information beyond what is provided by readily available physiological variables (19).

From our analysis, other CT features did not result as independent predictors of mortality. However, there are further considerations that are worth to be made. In particular, we believe that some CT features should be further investigated, as the results are available in one study only and/or the features could be investigated more in detail.

Firstly, in the overall SSc population, a CPFE pattern, namely the presence of emphysema together with ILD, is considered a mortality risk factor (9). However, among patients with SSc-ILD, CPFE did not resulted an independent predictor of shorter survival (10), while it may be considered a risk factor in ILD related to rheumatoid arthritis (27) and idiopathic pulmonary fibrosis (28). Interestingly, the presence of pulmonary hypertension was significantly associated with CPFE (44% patients with CPFE, 11% other ILD subjects, *p* < 0.0001) in one study (10), resulting an independent predictor of mortality. In our opinion, this suggests that the role of CPFE in SSc-ILD mortality could be deeper investigated, for example, considering also the extent of disease (i.e., of both emphysema and ILD) in comparison with ILD without emphysema. Moreover, taking into consideration also other conditions that may manifest in SSc-ILD, pleuroparenchymal fibroelastosis (PPFE) can be more common in SSc than in other CTD (29) and proved to determine a worse survival among SSc patients, irrespectively to its extent (30). However, the burden of PPFE only among SSC-ILD was not evaluated and should be addressed. Lastly, since CPFE and PPFE may be associated with different fibrotic patterns (28, 29), the influence of different associated ILD patterns could also be taken into consideration.

Then, the presence of an UIP pattern did not independently predict mortality in SSc-ILD (21). However, recently, the paper of Okamoto et al. (25), which was excluded from the analysis for some overlap patients, reported that UIP was a significant predictor of mortality on univariate analysis (a multivariate analysis was not performed), thus more data are desirable to confirm or reject UIP as a mortality predictor, preferably considering also the extent of disease. Moreover, in our review we did not find a comparison between UIP and NSIP in SSc-ILD. In fact, Takei et al. (21) compared UIP with all the other possible ILD patterns. A further selection of patients, based on the comparison between UIP and NSIP could be performed, as already suggested by Winstone et al. (7), since UIP is generally considered with worse prognosis while NSIP is the most common ILD pattern in SSc (6).

Lastly, bronchiectasis severity was investigated only in one study, resulting a predictor of mortality on univariate analysis but not confirmed as an independent prognostic factor (21). Bronchiectasis proved to be an independent risk factor for mortality in one out of two studies investigating patients with CTD-ILD (24, 26). Moreover, traction bronchiectasis inside GGO resulted as a predictor of mortality on multivariate analysis in one study on SSc-ILD (but with some overlaps) (25). Thus, we believe that also this CT feature need to be further investigated.

**CT features as predictors of ILD progression** were only analyzed on univariate analysis, with ILD (13, 16) and reticulation extent (16) resulting as significant risk factors for functional decline. Despite this, lacking a multivariate analysis, they cannot be confirmed as independent predictors. Moreover, CT features seem to be unable to predict ILD worsening on CT (15). Nevertheless, we have to note that only 3 studies investigated ILD progression (FVC decline or ILD increment on CT). Furthermore, we have also to specify that our review included only papers with a given definition of FVC decline or radiologic worsening, though accepting whatever formulation. We decided this criterion on a clinical basis: in fact, although statistically different trends of ILD progression of functional decline have been described and put in relation with CT features (10, 31–33), this may not represent a clinically meaningful worsening in pulmonary function or CT ILD features.

Our systematic review presents limitations, such as the variable methodologic quality of the included studies. Moreover, the heterogeneity of these studies makes it difficult to sum up, compare and discuss the whole data. Lastly, most predictors were evaluated only in one study.

## Conclusions

ILD extent and, in particular, extensive ILD are CT mortality predictors in SSc-ILD. Extensive ILD may be also considered a predictor of death, need for supplemental oxygen, or lung transplantation, as a combined outcome. ILD quantitative analysis parameters, fibrosis extent and reticulation extent seem also able to provide prognostic information on mortality risk, as well as honeycombing extent for respiratory mortality, but further studies are desirable to confirm these data. The role of CT features in predicting ILD progression remains to be further investigated.

## Data Availability Statement

The original contributions presented in the study are included in the article/ Supplementary Material, further inquiries can be directed to the corresponding author/s.

## Author Contributions

NL conceived the idea, planned the work, screened the articles, assessed the studies' quality, extrapolated and collected data and drafted the first version of the manuscript. SC and MM-C conceived the idea, planned the work and edited the final version of the manuscript. EC, CN, LC, GM, and ST checked data collection and edited the final version of the manuscript. CB conceived the idea, planned the work, resolved the disagreements on studies' selection as third assessor and edited the final version of the manuscript. MO conceived the idea, planned the work, screened the articles, assessed the studies' quality, collected data and edited the final version of the manuscript. All authors contributed to the article and approved the submitted version.

## Conflict of Interest

The authors declare that the research was conducted in the absence of any commercial or financial relationships that could be construed as a potential conflict of interest.

## Publisher's Note

All claims expressed in this article are solely those of the authors and do not necessarily represent those of their affiliated organizations, or those of the publisher, the editors and the reviewers. Any product that may be evaluated in this article, or claim that may be made by its manufacturer, is not guaranteed or endorsed by the publisher.

## Supplementary Material

Supplementary Figure 1 Risk of bias and applicability concerns, based on QUADAS-2 (http://www.bristol.ac.uk/population-health-sciences/projects/quadas/quadas-2/).

Supplementary Data 1 Papers not included (only partially meeting inclusion criteria), but relevant for the discussion, grouped by reasons of rejection.

Supplementary Data 2 Definitions of investigated predictors.

Supplementary Table 1 Search strategy.

Supplementary Table 2 Study quality assessment, based on the Critical Appraisal Skill Program questionnaire for cohort studies (http://www.casp-uk.net).

Supplementary Table 3 Predictors of mortality in ILD-SSc, multivariate analysis.

## Abbreviations

CPFE: combined pulmonary fibrosis emphysema
CTD: connective tissue disease
CT: chest computed tomography
GGO: ground glass opacities
FVC: forced vital capacity
ILD: interstitial lung disease
NSIP: non-specific interstitial pneumonia
SSc: systemic sclerosis
UIP: usual interstitial pneumonia.
