# Supplementary figures and images for: Computed Tomography Predictors of Mortality or Disease Progression in Systemic Sclerosis–Interstitial Lung Disease: A Systematic Review
Source: Front Med (Lausanne). 2022 Jan 27;8:807982. doi: 10.3389/fmed.2021.807982 (PMC8829727; doi:10.3389/fmed.2021.807982)

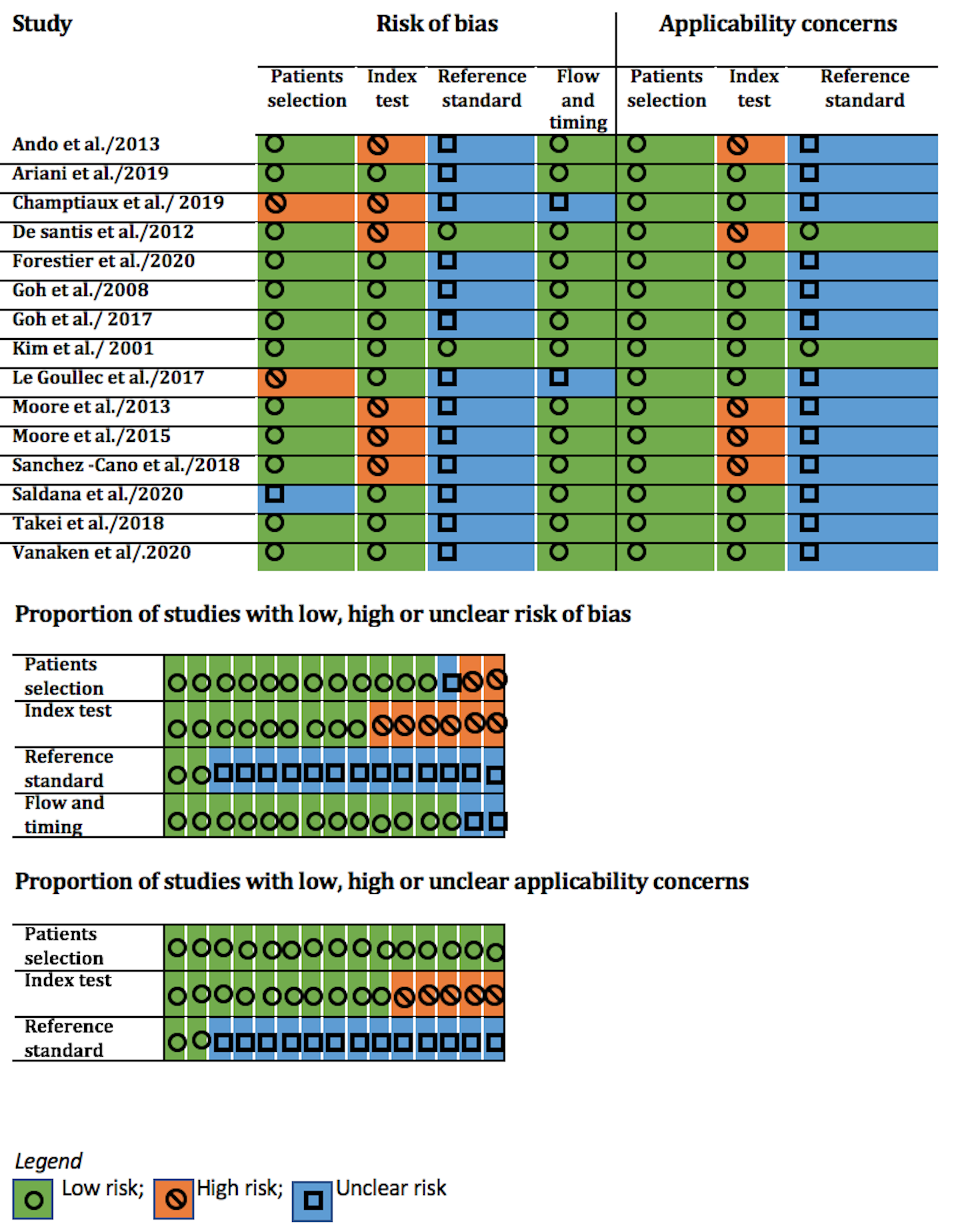

Supplement: Supplementary Figure 1 — Risk of bias and applicability concerns, based on QUADAS-2 (http://www.bristol.ac.uk/population-health-sciences/projects/quadas/quadas-2/). [file Image_1.TIFF]
