# Supplementary material for: Computed Tomography Predictors of Mortality or Disease Progression in Systemic Sclerosis–Interstitial Lung Disease: A Systematic Review
Source: Front Med (Lausanne). 2022 Jan 27;8:807982. doi: 10.3389/fmed.2021.807982 (PMC8829727; doi:10.3389/fmed.2021.807982)
Supplement: Supplementary Data 2 — Definitions of investigated predictors. [file Data_Sheet_2.docx]

**Supplementary Data 2**

**Definitions of investigated predictors**

***Predictors of mortality***

The CT features that were investigated as possible predictors among the various papers were:

- **Combined pulmonary fibrosis-emphysema** presence was evaluated in 2 studies and identified as presence of both ILD and emphysema with and extent ≥1% total lung parenchyma^1^ or as the presence of ILD and emphysema^2^.

**- Usual interstitial pneumonia** presence was evaluated in one manuscript^3^: UIP was defined according to the guidelines established by the American Thoracic Society, the European Respiratory Society, the Japanese Respiratory Society and the Latin American Thoracic Association described in 2011^4^.

- **ILD extent** was evaluated in one study^5^ scoring CT at 5 levels to the nearest 5%. Different extent thresholds were also tested (5%, 10%, 15%, 20%, 25%, 30%, 35%, 40%).

**- Extensive ILD,** in 6 works^5–10^, was defined as 1) ILD extent >30% or ranging from 10 to 30% with a FVC <70% predicted or 2) ILD extent >20% or =20% with a FVC <70% predicted. To identify extensive ILD, HRCT were scored, to the nearest 5%, at five levels in 4 works^5,6,8,9^, while at six levels in one^7^. One paper did not specify the scoring system^10^.

**- ILD grade** was assessed in one work^10^ as a score 1-4 , from GGO only to the presence of honeycombing.

- **ILD densitometric analysis** was performed in one study^11^, obtaining the following parameters by software analysis: mean lung attenuation (MLA), skewness, kurtosis, and the percentage of high attenuation areas (HAA). The change between two CT of those parameters for each patient (Δ) was also computed.

- **Fibrosis extent** was analysed in one paper^3^ and defined as the mean percentage of lung involvement due to reticulation and/or honeycombing, computed on six levels. Different thresholds of lung involvement were firstly tested, to define the best cut-off as mortality predictor.

- **Ground glass opacities (GGO) presence** was evaluated in one work^12^. The authors didn’t define the alteration.

- **GGO proportion**, expressed as the GGO % of the total ILD extent, was evaluated in one study^5^ at five levels, to the nearest 5%,.

**- GGO extent** was analysed in one paper, evaluated as a score 0-5 for each lung lobe^14^.

- **Reticulation presence** was evaluated in one work^12^. The authors didn’t define the alteration.

**- Reticulation coarseness** was assessed in one study^5^, evaluated at 5 levels as a score 0-3, from ground glass appearance to macrocystic honeycombing (air spaces greater than 4mm in diameter). The total score was the sum of each level’s score.

**- Reticulation extent** was computed in one paper^5^, evaluated at five levels, to nearest 5%. Also, four thresholds of the total amount of reticulation were investigated: 5%, 10%, 15%, 20%.

- **Honeycombing presence** was assessed in three works^5,14,15^, defined as clustered cystic airspaces from several millimetres to 1 cm in size with well-defined, thick walls in the subpleural regions in one paper^15^ or not defined^5,14^. One work evaluated the **presence of** **bilateral honeycombing**, too^14^.

**- Honeycombing extent** was assessed in one studies^14,16^, as the mean scores computed for each lung lobe (0-5 for each lobe)^14^.

- **Traction bronchiectasis severity** was evaluated in one work^3^ and scored from none to severe (0 to 3) based on the most severely affected airways, at five levels. The final score was computed by the mean score among the five levels.

***Predictors of ILD progression***

**- ILD extent** was evaluated in two study^5,13^ scoring CT at five^5^ or six levels^13^ to the nearest 5%.

- **Extensive ILD,** in 1 work^13^, was defined as ILD extent >20% and ILD=20% with a FVC <70% predicted. To identify extensive ILD, HRCT were scored, to the nearest 5%, at five levels.

**- ILD grade** was assessed in one work^13^ as a score 1-3 on the whole scan, from predominant GGO to predominant reticulation.

**- Fibrosis coarseness** was evaluated in one study^13^, as a score 0-3, from GGO alone to the presence of a macrocystic reticular pattern comprising air spaces larger than 4 mm in diameter. The total score was the sum of each level’s score.

- **GGO proportion**, expressed as the GGO % of the total ILD extent, was evaluated in two study^5,13^, evaluated at five^5^or six levels^13^.

**- GGO extent** was analysed in one paper^14^, evaluated to the nearest 5%, at five levels.

**- Irregular linear opacities extent** was computed in one work^16^, scored at five levels to the nearest 5%.

**- Reticulation proportion** was computed in one work^13^, as proportion of reticulation in the total ILD extent, at six levels.

**- Reticulation coarseness** was assessed in one study^5^, evaluated at 5 levels as a score 0-3, from ground glass appearance to macrocystic honeycombing (air spaces greater than 4mm in diameter).

**- Reticulation extent** was computed in one paper^5^, evaluated at five levels to ne nearest 5%.

- **Honeycombing presence** was assessed in two works^5,16^, not defined^6^ or defined as subpleural clustered cystic air spaces with distinct walls (>1 mm in thickness, often thick) of 3–25 mm in diameter^16^.

- **Traction bronchiectasis severity** was evaluated in one work and scored from none to severe (0 to 3) based on the most severely affected airways, at six levels^13^. The final score was the sum of the scores.

**- Emphysema extent** was evaluated in one study^13^ to the nearest 5% at six levels.

**References**

1. Ariani A, Silva M, Bravi E, Parisi S, Saracco M, De Gennaro F et al. Overall mortality in combined pulmonary fibrosis and emphysema related to systemic sclerosis. *RMD Open*. (2019) 5(1): e000820. doi:10.1136/rmdopen-2018-000820

2. Champtiaux N, Cottin V, Chassagnon G, Chaigne B, Valeyre D, Nunes H et al. Combined pulmonary fibrosis and emphysema in systemic sclerosis: A syndrome associated with heavy morbidity and mortality. *Semin Arthritis Rheum*. (2019) 49(1):98-104. doi: 10.1016/j.semarthrit.2018.10.011

3. Takei R, Arita M, Kumagai S, Ito Y, Tokioka F, Koyama T et al. Radiographic fibrosis score predicts survival in systemic sclerosis-associated interstitial lung disease: Radiographic fibrosis in SSc-ILD. *Respirology.* (2018) 23(4):385-391. doi:10.1111/resp.13175

4. Raghu G, Collard HR, Egan JJ, Martinez FJ, Behr J, Brown KK

et al. An Official ATS/ERS/JRS/ALAT Statement: Idiopathic Pulmonary Fibrosis: Evidence-based Guidelines for Diagnosis and Management. *Am J Respir Crit Care Med.* (2011) 183(6):788-824. doi:10.1164/rccm.2009-040GL

5. Goh NS, Desai SR, Veeraraghavan S, Hansell DM, Copley SJ, Maher TM et al. Interstitial Lung Disease in Systemic Sclerosis: A Simple Staging System. *Am J Respir Crit Care Med*. (2008) 177(11):1248-1254. doi:10.1164/rccm.200706-877OC

6. Goh NS, Hoyles RK, Denton CP, Hansell DM, Renzoni EA, Maher TM et al. Short-Term Pulmonary Function Trends Are Predictive of Mortality in Interstitial Lung Disease Associated with Systemic Sclerosis. *Arthritis Rheumatol*. (2017) 69(8):1670-1678. doi:10.1002/art.40130

7. Forestier A, Le Gouellec N, Béhal H, Kramer G, Perez T, Sobanski V et al. Evolution of high-resolution CT-scan in systemic sclerosis-associated interstitial lung disease: Description and prognosis factors. *Semin Arthritis Rheum*. (2020) 50(6):1406-1413. doi: 10.1016/j.semarthrit.2020.02.015

8. Moore OA, Goh N, Corte T, Rouse H, Hennessy O, Thakkar V et al. Extent of disease on high-resolution computed tomography lung is a predictor of decline and mortality in systemic sclerosis-related interstitial lung disease. *Rheumatology*. (2013) 52(1):155-160. doi:10.1093/rheumatology/kes289

9. Moore OA, Proudman SM, Goh N, Corte TJ, Rouse H, Hennessy O et al. Quantifying change in pulmonary function as a prognostic marker in systemic sclerosis-related interstitial lung disease. Clin *Exp Rheumatol.* (2015) 33(4 Suppl 91): S111-S116

10. Vanaken L, Landini N, Lenaerts J,  Claeys E, Lenaerts J, Wuyts WA et al. Progressive lung fibrosis and mortality can occur in early systemic sclerosis patients without pulmonary abnormalities at baseline assessment. *Clin Rheumatol*. (2020) 39(11):3393-3400. doi:10.1007/s10067-020-05105-4

11. Saldana DC, Hague CJ, Murphy D, Coxson HO, Tschirren J, Peterson S et al. Association of Computed Tomography Densitometry with Disease Severity, Functional Decline, and Survival in Systemic Sclerosis-associated Interstitial Lung Disease. *Ann Am Thorac Soc*. (2020) 17(7):813-820. doi:10.1513/AnnalsATS.201910-741OC

12. Sánchez-Cano D, Ortego-Centeno N, Callejas JL, Fonollosa Plá V, Ríos-Fernández R, Tolosa-Vilella C et al. Interstitial lung disease in systemic sclerosis: data from the spanish scleroderma study group. *Rheumatol Int.* (2018) 38(3):363-374. doi:10.1007/s00296-017-3916-x

13. Le Gouellec N, Duhamel A, Perez T, Hachulla AL, Sobanski V, Faivre JB et al. Predictors of lung function test severity and outcome in systemic sclerosis-associated interstitial lung disease. Kuwana M, ed. *PLOS ONE*. (2017) 12(8): e0181692. doi: 10.1371/journal.pone.0181692

14. De Santis M, Bosello SL, Peluso G, Pinnelli M, Alivernini S, Zizzo G et al. Bronchoalveolar lavage fluid and progression of scleroderma interstitial lung disease: Scleroderma interstitial lung disease. *Clin Respir J*. (2012) 6(1):9-17. doi:10.1111/j.1752-699X.2010. 00228.x

15. Ando K, Motojima S, Doi T, Nagaoka T, Kaneko N, Aoshima M et al. Effect of glucocorticoid monotherapy on pulmonary function and survival in Japanese patients with scleroderma-related interstitial lung disease. *Respir Investig*. (2013) 51(2):69-75. doi: 10.1016/j.resinv.2012.12.002

16. Kim EA, Johkoh T, Lee KS, Ichikado K, Koh EM, Kim TS et al. Interstitial Pneumonia in Progressive Systemic Sclerosis: Serial High-Resolution CT Findings with Functional Correlation: *J Comput Assist Tomogr*. (2001) 25(5):757-763. doi:10.1097/00004728-200109000-00015
