# Supplementary material for: Computed Tomography Predictors of Mortality or Disease Progression in Systemic Sclerosis–Interstitial Lung Disease: A Systematic Review
Source: Front Med (Lausanne). 2022 Jan 27;8:807982. doi: 10.3389/fmed.2021.807982 (PMC8829727; doi:10.3389/fmed.2021.807982)
Supplement: Supplementary Table 1 — Search strategy. [file Table_1.docx]

**Supplementary Table 1. Search strategy**

| **Row** | **Search terms^** | **Medline** | **Embase** | **Web of Science** |
| --- | --- | --- | --- | --- |
| **1** | 'scleroderma, limited'/exp OR 'scleroderma, diffuse'/exp OR 'scleroderma, systemic'/exp OR 'scleroderma, localized'/exp OR scleroderma OR 'systemic sclerosis' | 30,622 | 51,527 | 33,805 |
| **2** | 'interstitial lung diseases'/exp OR 'pulmonary fibrosis'/exp OR 'interstitial lung disease' OR 'pulmonary fibrosis' OR 'lung fibrosis' OR 'fibrosing alveolitis' OR ‘interstitial pneumon*’ OR ‘organizing pneumoni*’ OR ‘organising pneumoni*’ OR ‘combined pulmonary fibrosis’ OR ‘pleuroparenchymal fibroelastosis’ OR ‘diffuse alveolar damage’ OR ILD OR UIP OR NSIP OR OP OR CPFE OR PPFE OR OP OR DAD | 132,616 | 253,180 | 158,709 |
| **3** | prognos* OR progress* OR mortalit* OR death OR survival OR decline OR progression OR 'pulmonary function' OR 'lung function' OR PFT OR FVC OR 'vital capacity' OR VC OR TLC OR 'total lung capacity' | 4,566,361 | 6,026,270 | 5,136,292 |
| **4** | CT OR HRCT OR 'computed tomograph*' OR radiograph* OR radiologic* OR 'fibrosis score' OR 'fibrotic score’ | 1,428,960 | 2,053,772 | 1,555,825 |
| **5** | 1 and 2 and 3 | 669 | 1,982 | 862 |

**^** Terms shown with EMBASE syntax. Search field was “all fields”. Each term was adapted to each database.
