# Supplementary material for: Computed Tomography Predictors of Mortality or Disease Progression in Systemic Sclerosis–Interstitial Lung Disease: A Systematic Review
Source: Front Med (Lausanne). 2022 Jan 27;8:807982. doi: 10.3389/fmed.2021.807982 (PMC8829727; doi:10.3389/fmed.2021.807982)
Supplement: Supplementary Table 2 — Study quality assessment, based on the Critical Appraisal Skill Program questionnaire for cohort studies (http://www.casp-uk.net). [file Table_2.docx]

**Supplementary Table 2.** Study quality assessment, based on the Critical Appraisal Skill Program questionnaire for cohort studies (<http://www.casp-uk.net>)

**a)**

| **Quality questions and Study/Year** | **Ando et al./2013**^1^ | **Ariani et al./2019**^2^ | **Champtiaux et al./ 2019**^3^ | **De santis et al./2012**^4^ | **Forestier et al./2020**^5^ | **Goh et al./2008**^6^ | **Goh et al./ 2017**^7^ |
| --- | --- | --- | --- | --- | --- | --- | --- |
| **1.Did the study address a clearly focused issue?** | 1 | 1 | 1 | 1 | 1 | 1 | 1 |
| **2. Was the cohort recruited in an acceptable way?** | ? | 1 | 1 | 1 | 1 | 1 | 1 |
| **3. Was the ILD-SSC diagnosis accurately measured to minimize bias?** | 1 | 1 | 1 | 1 | 1 | 1 | 1 |
| **4. Was the mortality or ILD progression accurately measured to minimize bias?** | 1 | 1 | 1 | 1 | 1 | 1 | 1 |
| **5a. Have the authors identified all important confounding factors?** | ? | x | x | x | 1 | 1 | 1 |
| **5b. Have they taken into account of the confounding factors in the design and/or analysis?** | ? | x | x | x | ? | 1 | 1 |
| **6. (a) Was the follow up of subjects complete enough?** | 1 | 1 | 1 | 1 | 1 | 1 | 1 |
| **6. (b) Was the follow up of subjects long enough?** | ? | ? | ? | ? | ? | ? | 1 |
| **7. What are the results of this study?** | 1 | 1 | x | 1 | 1 | 1 | 1 |
| **8. How precise are the results?** | 1 | 1 | x | 1 | 1 | 1 | 1 |
| **9. Do you believe the results?** | 1 | 1 | 1 | 1 | 1 | 1 | 1 |
| **10. Can the results be applied to the local population?** | 1 | 1 | 1 | 1 | 1 | 1 | 1 |
| **11. Do the results of this study fit with other available evidence?** | 1 | x | x | 1 | 1 | 1 | 1 |

**b)**

| **Quality questions and Study/Year** | **Kim et al./ 2001**^8^ | **Le Goullec et al./2017**^9^ | **Moore et al./2013**^10^ | **Moore et al./2015**^11^ | **Sanchez -Cano et al./2018**^12^ | **Saldana et al./2020**^13^ | **Takei et al./2018**^14^ | **Vanaken et al/.2020**^15^ |
| --- | --- | --- | --- | --- | --- | --- | --- | --- |
| **1.Did the study address a clearly focused issue?** | 1 | 1 | 1 | 1 | 1 | 1 | 1 | 1 |
| **2. Was the cohort recruited in an acceptable way?** | 1 | ? | 1 | 1 | 1 | 1 | 1 | 1 |
| **3. Was the ILD-SSC diagnosis accurately measured to minimize bias?** | 1 | 1 | 1 | 1 | x | ? | 1 | 1 |
| **4. Was the mortality or ILD progression accurately measured to minimize bias?** | 1 | 1 | 1 | 1 | 1 | 1 | 1 | 1 |
| **5a. Have the authors identified all important confounding factors?** | 1 | 1 | x | ? | x | 1 | 1 | 1 |
| **5b. Have they taken into account of the confounding factors in the design and/or analysis?** | ? | x | x | ? | x | 1 | 1 | 1 |
| **6. (a) Was the follow up of subjects complete enough?** | 1 | 1 | 1 | 1 | 1 | 1 | 1 | 1 |
| **6. (b) Was the follow up of subjects long enough?** | ? | 1 | ? | ? | ? | ? | 1 | 1 |
| **7. What are the results of this study?** | 1 | 1 | 1 | 1 | 1 | 1 | 1 | 1 |
| **8. How precise are the results?** | 1 | 1 | 1 | 1 | 1 | 1 | 1 | 1 |
| **9. Do you believe the results?** | 1 | 1 | 1 | 1 | 1 | 1 | 1 | 1 |
| **10. Can the results be applied to the local population?** | 1 | 1 | 1 | 1 | 1 | 1 | 1 | 1 |
| **11. Do the results of this study fit with other available evidence?** | 1 | 1 | 1 | 1 | x | 1 | ? | ? |

1: appropriate study design as it pertains to the question; x inadequate or incomplete methods; ? uncertainty

**References**

1. Ando K, Motojima S, Doi T, Nagaoka T, Kaneko N, Aoshima M et al. Effect of glucocorticoid monotherapy on pulmonary function and survival in Japanese patients with scleroderma-related interstitial lung disease. *Respir Investig*. (2013) 51(2):69-75. doi: 10.1016/j.resinv.2012.12.002

2. Ariani A, Silva M, Bravi E, Parisi S, Saracco M, De Gennaro F et al. Overall mortality in combined pulmonary fibrosis and emphysema related to systemic sclerosis. *RMD Open*. (2019) 5(1): e000820. doi:10.1136/rmdopen-2018-000820

3. Champtiaux N, Cottin V, Chassagnon G, Chaigne B, Valeyre D, Nunes H et al. Combined pulmonary fibrosis and emphysema in systemic sclerosis: A syndrome associated with heavy morbidity and mortality. *Semin Arthritis Rheum*. (2019) 49(1):98-104. doi: 10.1016/j.semarthrit.2018.10.011

4. De Santis M, Bosello SL, Peluso G, Pinnelli M, Alivernini S, Zizzo G et al. Bronchoalveolar lavage fluid and progression of scleroderma interstitial lung disease: Scleroderma interstitial lung disease. *Clin Respir J*. (2012) 6(1):9-17. doi:10.1111/j.1752-699X.2010. 00228.x

5. Forestier A, Le Gouellec N, Béhal H, Kramer G, Perez T, Sobanski V et al. Evolution of high-resolution CT-scan in systemic sclerosis-associated interstitial lung disease: Description and prognosis factors. *Semin Arthritis Rheum*. (2020) 50(6):1406-1413. doi: 10.1016/j.semarthrit.2020.02.015

6. Goh NS, Desai SR, Veeraraghavan S, Hansell DM, Copley SJ, Maher TM et al. Interstitial Lung Disease in Systemic Sclerosis: A Simple Staging System. *Am J Respir Crit Care Med*. (2008) 177(11):1248-1254. doi:10.1164/rccm.200706-877OC

7. Goh NS, Hoyles RK, Denton CP, Hansell DM, Renzoni EA, Maher TM et al. Short-Term Pulmonary Function Trends Are Predictive of Mortality in Interstitial Lung Disease Associated with Systemic Sclerosis. *Arthritis Rheumatol*. (2017) 69(8):1670-1678. doi:10.1002/art.40130

8. Kim EA, Johkoh T, Lee KS, Ichikado K, Koh EM, Kim TS et al. Interstitial Pneumonia in Progressive Systemic Sclerosis: Serial High-Resolution CT Findings with Functional Correlation: *J Comput Assist Tomogr*. (2001) 25(5):757-763. doi:10.1097/00004728-200109000-00015

9. Le Gouellec N, Duhamel A, Perez T, Hachulla AL, Sobanski V, Faivre JB et al. Predictors of lung function test severity and outcome in systemic sclerosis-associated interstitial lung disease. Kuwana M, ed. *PLOS ONE*. (2017) 12(8): e0181692. doi: 10.1371/journal.pone.0181692

10. Moore OA, Goh N, Corte T, Rouse H, Hennessy O, Thakkar V et al. Extent of disease on high-resolution computed tomography lung is a predictor of decline and mortality in systemic sclerosis-related interstitial lung disease. *Rheumatology*. (2013) 52(1):155-160. doi:10.1093/rheumatology/kes289

11. Moore OA, Proudman SM, Goh N, Corte TJ, Rouse H, Hennessy O et al. Quantifying change in pulmonary function as a prognostic marker in systemic sclerosis-related interstitial lung disease. Clin *Exp Rheumatol.* (2015) 33(4 Suppl 91): S111-S116

12. Sánchez-Cano D, Ortego-Centeno N, Callejas JL, Fonollosa Plá V, Ríos-Fernández R, Tolosa-Vilella C et al. Interstitial lung disease in systemic sclerosis: data from the spanish scleroderma study group. *Rheumatol Int.* (2018) 38(3):363-374. doi:10.1007/s00296-017-3916-x

13. Saldana DC, Hague CJ, Murphy D, Coxson HO, Tschirren J, Peterson S et al. Association of Computed Tomography Densitometry with Disease Severity, Functional Decline, and Survival in Systemic Sclerosis-associated Interstitial Lung Disease. *Ann Am Thorac Soc*. (2020) 17(7):813-820. doi:10.1513/AnnalsATS.201910-741OC

14. Takei R, Arita M, Kumagai S, Ito Y, Tokioka F, Koyama T et al. Radiographic fibrosis score predicts survival in systemic sclerosis-associated interstitial lung disease: Radiographic fibrosis in SSc-ILD. *Respirology.* (2018) 23(4):385-391. doi:10.1111/resp.13175

15. Vanaken L, Landini N, Lenaerts J,  Claeys E, Lenaerts J, Wuyts WA et al. Progressive lung fibrosis and mortality can occur in early systemic sclerosis patients without pulmonary abnormalities at baseline assessment. *Clin Rheumatol*. (2020) 39(11):3393-3400. doi:10.1007/s10067-020-05105-4
