# Supplementary material for: Computed Tomography Predictors of Mortality or Disease Progression in Systemic Sclerosis–Interstitial Lung Disease: A Systematic Review
Source: Front Med (Lausanne). 2022 Jan 27;8:807982. doi: 10.3389/fmed.2021.807982 (PMC8829727; doi:10.3389/fmed.2021.807982)
Supplement: Supplementary Table 3 — Predictors of mortality in ILD-SSc, multivariate analysis. [file Table_3.docx]

**Supplementary Table 3. Predictors of mortality in ILD-SSc, multivariate analysis.**

| **Predictor and Study/Year** | **Outcome** | **Hazard ratio** | **Confidence interval** | **p-value** | **Variables** | **Independent**  **predictor** |
| --- | --- | --- | --- | --- | --- | --- |
| **CPFE** |  |  |  |  |  |  |
| Champtiaux, et al./ 2019^1^ | Survival | - | - | 0.13 | N/R | no |
| **ILD extent** |  |  |  |  |  |  |
| Goh et al./2008^2^ | Mortality | **1.03** | **1.02-1.05** | **<0.005** | Age, sex, smoking status, ground glass proportion, reticulation coarseness | **yes** |
| **Extensive ILD** |  |  |  |  |  |  |
| Goh et al./2008^2$^ | Mortality | **3.66** | **2.25-5.97** | **<0.005** | Age, sex, smoking status, PH | **yes** |
| Goh et al./2008^2$a^ | Mortality | **3.02** | **1.46-6.27** | **<0.005** | Age, sex, smoking status, PH | **yes** |
| Goh et al./2008^2$b^ | Mortality | **3.76** | **1.18-4.34** | **<0.005** | Age, sex, smoking status, PH | **yes** |
| Goh et al./2008^§ù^ | Mortality | **3.39 to**  **3.82** | **1.51-7.61**  **2.03-7.19** | **<0.005** | Age, sex, smoking status, PH | **yes** |
| Moore et al./2013^3§^ | Death, need for supplemental oxygen or lung transplantation | **3.00** | **1.20-7.51** | **0.02** | FVC, DLCO/VA, age at HRCT, ATA | **yes** |
| Moore et al./2013^3§^** | Death, need for supplemental oxygen or lung transplantation | **3.30** | **1.10-9.93** | **0.03** | FVC, DLCO/VA, age at PFT, PH, immunosuppressant therapy, ATA | **yes** |
| Goh et al./ 2017^4ç^ | 15 y survival | **3.01** | **1.90-4.74** | **<0.0005** | FVC, DLCO, FVC/DLCO, KCO, age, sex, treatment status, smoking status, ATA, cutaneous involvement | **yes** |
| Goh et al./ 2017^4ç^ | 15 y survival | **2.30** | **1.43-3.70** | **<0.001** | KCO, KCO decline°, CCD, age, sex, treatment status, smoking status, PH, disease duration^#^ | **yes** |
| Goh et al./ 2017^4ç^ | 15 y survival | **2.76** | **1.66-4.80** | **<0.0005** | KCO, KCO decline§, CCD, age, sex, treatment status, smoking status, PH, disease duration^ | **yes** |
| **ILD quantitative analysis** |  |  |  |  |  |  |
| Saldana et al./2020^5^  (HAA %) | Mortality | 1.19 | 0.99-1.43 | 0.07 | age, sex, pack-years, race | no |
| Saldana et al./2020^5^  (Skewness) | Mortality | **0.15** | **0.03-0.84** | **0.03** | age, sex, pack-years, race | **yes** |
| Saldana et al./2020^5^  (Kurtosis) | Mortality | **0.65** | **0.46-0.94** | **0.02** | age, sex, pack-years, race | **yes** |
| Saldana et al./2020^5^  (MLA HU) | Mortality | 1.01 | 1.00-1.02 | 0.07 | age, sex, pack-years, race | no |
| Saldana et al./2020^5^  (∆ HAA %) | Mortality | **1.37** | **1.02-1.83** | **0.03** | age, sex, pack-years, race | **yes** |
| Saldana et al./2020^5^  (∆ Skewness) | Mortality | 0.01 | 2.60^e-5^-5.34 | 0.15 | age, sex, pack-years, race | no |
| Saldana et al./2020^5^  (∆ Kurtosis) | Mortality | 0.47 | 0.11-2.05 | 0.32 | age, sex, pack-years, race | no |
| Saldana et al./2020^5^  (∆ MLA HU) | Mortality | **1.05** | **1.00-1.11** | **0.04** | age, sex, pack-years, race | **yes** |
| Saldana et al./2020^5^  (HAA %) | Mortality | 1.02 | 0.95-1.11 | 0.54 | age, sex, pack-years, visual fibrosis scores | no |
| Saldana et al./2020^5^  (Skewness) | Mortality | 0.50 | 0.23-1.11 | 0.09 | age, sex, pack-years, visual fibrosis scores | no |
| Saldana et al./2020^5^  (Kurtosis) | Mortality | 0.88 | 0.75-1.05 | 0.15 | age, sex, pack-years, visual fibrosis scores | no |
| Saldana et al./2020^5^  (MLA HU) | Mortality | 1.06 | 0.99-1.13 | 0.07 | age, sex, pack-years, visual fibrosis scores | no |
| Saldana et al./2020^5^  (∆ HAA %) | Mortality | **1.36** | **1.17-1.59** | **< 0.001** | age, sex, pack-years, visual fibrosis scores | **yes** |
| Saldana et al./2020^5^  (∆ Skewness) | Mortality | **0.04** | **0.01-0.31** | **0.002** | age, sex, pack-years, visual fibrosis scores | **yes** |
| Saldana et al./2020^5^  (∆ Kurtosis) | Mortality | **0.47** | **0.30-0.74** | **< 0.001** | age, sex, pack-years, visual fibrosis scores | **yes** |
| Saldana et al./2020^5^  (∆ MLA HU) | Mortality | **1.05** | **1.03-1.06** | **< 0.001** | age, sex, pack-years, visual fibrosis scores | **yes** |
| Saldana et al./2020^5^  (HAA %) | Mortality | 1.03 | 0.96- 1.11 | 0.41 | age, sex, pack-years, FVC, DLCO | no |
| Saldana et al./2020^5^  (Skewness) | Mortality | 0.79 | 0.36- 1.71 | 0.55 | age, sex, pack-years, FVC, DLCO | no |
| Saldana et al./2020^5^  (Kurtosis) | Mortality | 0.97 | 0.84- 1.13 | 0.70 | age, sex, pack-years, FVC, DLCO | no |
| Saldana et al./2020^5^  (MLA HU) | Mortality | 0.98 | 0.84- 1.13 | 0.74 | age, sex, pack-years, FVC, DLCO | no |
| Saldana et al./2020^5^  (∆ HAA %) | Mortality | 1.12 | 0.89- 1.41 | 0.32 | age, sex, pack-years, FVC, DLCO | no |
| Saldana et al./2020^5^  (∆ Skewness) | Mortality | 6.32 | 0.36- 110.18 | 0.21 | age, sex, pack-years, FVC, DLCO | no |
| Saldana et al./2020^5^  (∆ Kurtosis) | Mortality | 1.33 | 0.77- 2.32 | 0.31 | age, sex, pack-years, FVC, DLCO | no |
| Saldana et al./2020^5^  (∆ MLA HU) | Mortality | 1.01 | 0.98- 1.03 | 0.65 | age, sex, pack-years, FVC, DLCO | no |
| Saldana et al./2020^5^  (HAA %) | Mortality | 1.03 | 0.97- 1.09 | 0.34 | ILD-GAP Index | no |
| Saldana et al./2020^5^  (Skewness) | Mortality | 0.74 | 0.40- 1.35 | 0.33 | ILD-GAP Index | no |
| Saldana et al./2020^5^  (Kurtosis) | Mortality | 0.95 | 0.84- 1.06 | 0.35 | ILD-GAP Index | no |
| Saldana et al./2020^5^  (MLA HU) | Mortality | 1.02 | 0.97- 1.07 | 0.46 | ILD-GAP Index | no |
| Saldana et al./2020^5^  (∆ HAA %) | Mortality | **1.36** | **1.19- 1.55** | **< 0.001** | ILD-GAP Index | **yes** |
| Saldana et al./2020^5^  (∆ Skewness) | Mortality | **0.05** | **0.01- 0.32** | **< 0.001** | ILD-GAP Index | **yes** |
| Saldana et al./2020^5^  (∆ Kurtosis) | Mortality | **0.51** | **0.35- 0.76** | **< 0.001** | ILD-GAP Index | **yes** |
| Saldana et al./2020^5^  (∆ MLA HU) | Mortality | **1.04** | **1.02- 1.05** | **< 0.001** | ILD-GAP Index | **yes** |
| Saldana et al./2020^5^  (HAA %) | Mortality | 1.17 | 0.94- 1.45 | 0.17 | SADL model | no |
| Saldana et al./2020^5^  (Skewness) | Mortality | 0.67 | 0.37- 1.22 | 0.19 | SADL model | no |
| Saldana et al./2020^5^  (Kurtosis) | Mortality | 0.73 | 0.46- 1.17 | 0.19 | SADL model | no |
| Saldana et al./2020^5^  (MLA HU) | Mortality | 1.02 | 0.97- 1.08 | 0.35 | SADL model | no |
| Saldana et al./2020^5^  (∆ HAA %) | Mortality | **1.40** | **1.22- 1.61** | **< 0.001** | SADL model | **yes** |
| Saldana et al./2020^5^  (∆ Skewness) | Mortality | **0.04** | **0.01- 0.27** | **< 0.001** | SADL model | **yes** |
| Saldana et al./2020^5^  (∆ Kurtosis) | Mortality | **0.49** | **0.32- 0.74** | **< 0.001** | SADL model | **yes** |
| Saldana et al./2020^5^  (∆ MLA HU) | Mortality | **1.04** | **1.03- 1.06** | **< 0.001** | SADL model | **yes** |
| **Fibrosis extent** |  |  |  |  |  |  |
| Takei et al./2018^6^ | 5 y survival | **1.03** | **1.00-1.06** | **0.049** | Age, sex, CKD, FVC, TB | **yes** |
| **GGO extent** |  |  |  |  |  |  |
| De Santis et al./2012^7^ | Mortality | - | - | ≥0.05 | N/R | no |
| De Santis et al./2012^7^ | Respiratory mortality | - | - | ≥0.05 | N/R | no |
| **Reticulation extent** |  |  |  |  |  |  |
| Goh et al./2008^2^ | Mortality | **1.05** | **1.02-1.08** | **<0.001** | Age, sex, smoking status, proportion of ground glass, coarseness of reticulation | **yes** |
| **Honeycombing presence** |  |  |  |  |  |  |
| Ando et al./2013^8^ | Survival | 3.45 | 0.72-16.50 | 0.12 | FVC, PH | no |
| De Santis et al./2012^7^  (bilateral) | Mortality | - | - | ≥0.05 | N/R | no |
| De Santis et al./2012^7^  (bilateral) | Respiratory mortality | - | - | ≥0.05 | N/R | no |
| **Honeycombing extent** |  |  |  |  |  |  |
| De Santis et al./2012^7^ | Mortality | - | - | ≥0.05 | N/R | no |
| De Santis et al./2012^7^ | Respiratory mortality | **1.23** | **1.10-1.44** | **<0.05** | **N/R** | **yes** |
| **TB** |  |  |  |  |  |  |
| Takei et al./2018^6^ | 5 y Survival | N/R | N/R | ≥0.05 | Age, sex, CKD, FVC, TB | no |

^a^ Patients without immediate therapy

^b^ Patients with therapy initiated or continued at presentation

^$^ILD extent >30% or ILD ranging from 10 to 30% with a predicted FVC <70%

^§^ILD >20% or =20% with a predicted FVC <70%

^ù^data available on 108/215 patients

^ç^adopted Extensive ILD definition not stated

*corrected for CT disease extent changes over time (data available on 93/172 patients)

° at 12 months, ^§^ at 24 months

^#^data available on 149 patients

^data available on 142 patients

Bold Valuescorrespond to statistically significant predictors.

ATA: Anti-topoisomerase antibodies; CCD: composite categorical decline; FVC: forced vital capacity; CKD: chronic kidney failure; DLCO: Diffusion capacity for carbon monoxide; DLCO/VA: DLCO by alveolar volume ratio; FVC = forced vital capacity; GAP: gender, age, physiology; HAA, high attenuation areas; HU, Hounsfield unit; ILD: interstitial lung disease; KCO: carbon monoxide transfer coefficient; MLA, mean lung attenuation; N/R: not reported; PFT: pulmonary function tests; PH: pulmonary hypertension; SADL: smoking history, age, DLCO; SSc: systemic sclerosis; TB: traction bronchiectasis severity; y: years.

**References**

1. Champtiaux N, Cottin V, Chassagnon G, Chaigne B, Valeyre D, Nunes H et al. Combined pulmonary fibrosis and emphysema in systemic sclerosis: A syndrome associated with heavy morbidity and mortality. *Semin Arthritis Rheum*. (2019) 49(1):98-104. doi: 10.1016/j.semarthrit.2018.10.011

2. Goh NS, Desai SR, Veeraraghavan S, Hansell DM, Copley SJ, Maher TM et al. Interstitial Lung Disease in Systemic Sclerosis: A Simple Staging System. *Am J Respir Crit Care Med*. (2008) 177(11):1248-1254. doi:10.1164/rccm.200706-877OC

3. Moore OA, Goh N, Corte T, Rouse H, Hennessy O, Thakkar V et al. Extent of disease on high-resolution computed tomography lung is a predictor of decline and mortality in systemic sclerosis-related interstitial lung disease. *Rheumatology*. (2013) 52(1):155-160. doi:10.1093/rheumatology/kes289

4. Goh NS, Hoyles RK, Denton CP, Hansell DM, Renzoni EA, Maher TM et al. Short-Term Pulmonary Function Trends Are Predictive of Mortality in Interstitial Lung Disease Associated with Systemic Sclerosis. *Arthritis Rheumatol*. (2017) 69(8):1670-1678. doi:10.1002/art.40130

5. Saldana DC, Hague CJ, Murphy D, Coxson HO, Tschirren J, Peterson S et al. Association of Computed Tomography Densitometry with Disease Severity, Functional Decline, and Survival in Systemic Sclerosis-associated Interstitial Lung Disease. *Ann Am Thorac Soc*. (2020) 17(7):813-820. doi:10.1513/AnnalsATS.201910-741OC

6. Takei R, Arita M, Kumagai S, Ito Y, Tokioka F, Koyama T et al. Radiographic fibrosis score predicts survival in systemic sclerosis-associated interstitial lung disease: Radiographic fibrosis in SSc-ILD. *Respirology.* (2018) 23(4):385-391. doi:10.1111/resp.13175

7. De Santis M, Bosello SL, Peluso G, Pinnelli M, Alivernini S, Zizzo G et al. Bronchoalveolar lavage fluid and progression of scleroderma interstitial lung disease: Scleroderma interstitial lung disease. *Clin Respir J*. (2012) 6(1):9-17. doi:10.1111/j.1752-699X.2010. 00228.x

8. Ando K, Motojima S, Doi T, Nagaoka T, Kaneko N, Aoshima M et al. Effect of glucocorticoid monotherapy on pulmonary function and survival in Japanese patients with scleroderma-related interstitial lung disease. *Respir Investig*. (2013) 51(2):69-75. doi: 10.1016/j.resinv.2012.12.002
